# Cost-effectiveness of a corticosteroid injection versus exercise therapy for shoulder pain in general practice (SIX-Shoulder Study): a randomized controlled trial

**DOI:** 10.1093/fampra/cmaf081

**Published:** 2025-11-17

**Authors:** Annelotte H C Versloot, Mehlika Toy, Dieuwke Schiphof, Patrick Bindels, Ramon P G Ottenheijm, Marloes de Graaf, Daniëlle A van der Windt, John M van Ochten, Bart W Koes, Jos Runhaar

**Affiliations:** Department of General Practice, Erasmus MC University Medical Center Rotterdam, Rotterdam 3015 GD, The Netherlands; Department of Health Technology Assessment, Erasmus School of Health Policy & Management, Rotterdam 3062 PA, The Netherlands; Department of General Practice, Erasmus MC University Medical Center Rotterdam, Rotterdam 3015 GD, The Netherlands; Department of General Practice, Erasmus MC University Medical Center Rotterdam, Rotterdam 3015 GD, The Netherlands; Department of Family Medicine, Care and Public Health Research Institute (CAPHRI), Maastricht University, Maastricht 6211 LH, The Netherlands; Department of Manual Therapy, Breederode University of Applied Science, Rotterdam 3011 XA, The Netherlands; School of Medicine, Primary Care Centre Versus Arthritis, Keele University, Keele ST5 5BG, United Kingdom; Department of General Practice, Erasmus MC University Medical Center Rotterdam, Rotterdam 3015 GD, The Netherlands; Department of General Practice, Erasmus MC University Medical Center Rotterdam, Rotterdam 3015 GD, The Netherlands; Research Unit of General Practice, Department of Public Health & Center for Muscle & Joint Health, University of Southern Denmark, Odense 5230, Denmark; Department of General Practice, Erasmus MC University Medical Center Rotterdam, Rotterdam 3015 GD, The Netherlands

**Keywords:** musculoskeletal pain, musculoskeletal/connective tissue disorders, primary care, physical activity/exercise, health economics

## Abstract

**Background:**

Shoulder pain often results in functional limitations leading to substantial societal and healthcare costs. Guidelines recommend a corticosteroid injection or exercise therapy, but long-term comparative cost-effectiveness remains unclear. This study examined the cost-effectiveness of these two treatments over 12 months in patients with shoulder pain presenting in Dutch primary care.

**Methods:**

A randomized controlled trial was conducted with patients consulting for a new episode of shoulder pain. Participants were randomly assigned to a corticosteroid injection or a 12-week physiotherapist-led exercise therapy program. Participants completed questionnaires at baseline, 6 weeks, and 3, 6, 9, and 12 months. The primary outcome was incremental costs per quality-adjusted life year (QALY) gained over 12 months, analyzed with the incremental cost-effectiveness ratio (ICER). QALY was measured using the EuroQol Five-Dimensional Questionnaire (EQ-5D-5L) score.

**Results:**

A total of 183 participants were included, with 91 participants in the injection group and 92 in the exercise therapy group. The incremental costs and QALY for the exercise group were, respectively, €428 (95% CI: −1825 to 2682) and 0.02957 (95% CI: −0.0299 to 0.0891), resulting in an ICER of €14 489 (95% CI: −1 698 053 to 1 727 032) per QALY gained. With a willingness-to-pay threshold of 50 000 the cost-effectiveness acceptability curve showed a probability of 70% of cost-effectiveness for exercise therapy.

**Conclusions:**

For patients with shoulder pain, the exercise therapy group has a probability of 70% to be cost-effective compared to the injection group over a 12-month follow-up with an ICER of €14 489 (95% CI: −1 698 053 to 1 727 032) at a willingness-to-pay threshold of €50 000 per QALY.

**Clinical trial registration:**

registered in the Netherlands Trial Registry (NL-OMON52854).

Key messages

- This research investigated the cost-effectiveness of treatments for shoulder pain.
- The mean costs were higher in the exercise therapy group, but also the mean QALYs.
- There is a probability of 70% of cost-effectiveness for exercise therapy.
- 16.4% of the participants accounted for 74% of the costs.

## Introduction

Shoulder pain is the third most prevalent musculoskeletal complaint in primary healthcare, with an estimated incidence of 30.3 per 1000 person-years. The prognosis for shoulder pain is often poor; only 50% of individuals presenting with a new episode in primary care achieve full recovery within six months [1, 2]. The Clinical Knowledge Summaries of the National Institute for Health and Care Excellence (NICE) and the care pathways guidelines developed in collaboration with the UK National Health Service (NHS) state that the treatment of shoulder pain should focus on pain reduction and managing shoulder function [3, 4]. Therefore, the Dutch College of General Practitioners (DCGP) guideline for shoulder pain recommends a stepped care approach [5]. Initially, the GP provides advice and, if necessary, prescribes analgesics. Should the patient return with persisting complaints, the guideline advises to give a corticosteroid injection or a referral to physiotherapy. The guidelines, however, do not specify which treatment is to be preferred or considered more effective.

Besides pain, patients frequently experience functional limitations that can be severe enough to hinder work-related tasks. Literature shows that a high intensity of musculoskeletal pain is strongly associated with significant societal and healthcare costs due to work absence and treatment costs [6, 7]. A cost-estimation study in Sweden found the mean annual cost per patient consulting for shoulder pain in primary care to be €4139, with sick leave comprising over 80% of the total costs [8]. However, the study did not consider societal factors (such as productivity loss), and the data were gathered from electronic health records, which may lead to less complete results.

Given the high incidence of shoulder complaints, the lack of high-quality trials comparing exercise therapy with a corticosteroid injection, and the substantial societal and healthcare costs, the Shoulder Injection and Exercise Therapy (SIX) study was initiated. This randomized controlled trial (RCT) compared the clinical and cost-effectiveness of corticosteroid injections with physiotherapist-led exercise therapy for patients with shoulder pain in primary care. The objective of this study was to compare the cost-effectiveness of these two treatments for shoulder pain when offered in primary care over 12 months of follow-up. The clinical outcomes of this trial are reported elsewhere [9] (submitted for publication).

## Methods

### Design

This study was a randomized, multicenter, pragmatic clinical trial and conducted in accordance with the previously published protocol by van Doorn *et al.* [10], registered in the Netherlands Trial Registry (NL-OMON52854). This RCT was reported in accordance with the CONSORT guidelines [11].

### Recruitment and participants

There were 55 Dutch general practices in Rotterdam and the surrounding area participating in the study to recruit patients. Patients over 18 years old with shoulder pain who consulted their GP and were eligible for a corticosteroid injection and exercise therapy were invited for trial enrollment. Patients were not eligible if the shoulder pain was due to a recent or earlier trauma, neurological or cardiac disease, or instability of the joint. They were also not eligible if they already received a corticosteroid injection or physiotherapy for the shoulder in the past 6 months.

### Consent and randomization

After eligibility verification and patients had provided written informed consent, the baseline questionnaire was sent. After completing that questionnaire, participants were randomized to either the corticosteroid injection group or the exercise therapy group, using a computerized randomization scheme. The Erasmus MC Clinical Trial Center prepared a web-based randomization system using random blocks of 8, 6, or 4 to ensure concealment of allocation. Both the participants and the GP were notified of the randomization outcome. To ensure blinding, the researcher performing the analysis was blinded for treatment allocation.

After giving the injection or the referral for exercise therapy, the GP filled in a checklist, including details on the assigned physiotherapy practice or the injection site, and the GP's working diagnosis. A flow-chart detailing the recruitment and follow-up process is provided in Supplementary Appendix S1.

### Interventions

#### Corticosteroid injection

The corticosteroid injection consisted of 40 mg triamcinolone acetonide (Kenacort-A 40), possibly in combination with lidocaine 10 mg, in accordance with the DCGP guidelines for shoulder pain [5], and was administered by their treating GP. Based on the presentation of the shoulder condition, they administered the corticosteroid injection into either the glenohumeral or the subacromial space. In accordance with the guidelines, it was allowed to receive a second corticosteroid injection after 2 weeks if the shoulder pain persisted.

#### Exercise therapy

Participants assigned to exercise therapy were referred to local affiliated physiotherapists (*n* = 49), preferably part of the Dutch Shoulder Network (SNN), an umbrella organization with specialized physiotherapists. The therapy included up to 12 supervised sessions of 30 min each. All participating physiotherapists received a brief guideline, outlining the exercise therapy setup and treatment parameters (see Supplementary Appendix S2). This guideline was made in collaboration with SNN and based on the existing guideline for the treatment of shoulder pain for physiotherapists [12].

#### Co-interventions

It was permitted for participants to continue with their usual medication as discussed with their GP, since this was a pragmatic clinical trial. Additionally, co-interventions were allowed and tracked using questionnaires.

### Data collection

A total of six questionnaires were sent to the participants: at baseline, after 6 weeks, and after 3, 6, 9, and 12 months. The questionnaires were sent by e-mail to the participants using a web-based medical survey tracker (Gemstracker). If a participant preferred the questionnaire on paper, it was sent by post.

### Outcomes

The outcome of this study was incremental costs per quality-adjusted life year (QALY) gained, using both the Medical Consumption Questionnaire (MCQ) [13] and the Productivity Costs Questionnaire (PCQ) [14], over 12 months post-randomization. QALY was measured using the five-level version of the well-validated EuroQol Five-Dimensional Questionnaire (EQ-5D-5L) score [15].

#### Clinical variables

The following clinical outcomes were measured at baseline: the Shoulder Pain and Disability Index (SPADI) [16, 17], health-related quality of life using the EuroQol Five-Dimensional Questionnaire (EQ-5D-5L), sleep quality (Sleep Quality Scale), pain intensity using Visual Analogue Scale (VAS), Hospital Anxiety and Depression Scale (HADS) [18], and the Fear-Avoidance Beliefs Questionnaire (FABQ) [19]. The baseline questionnaire also included sociodemographic questions about age, BMI, current and past shoulder pain, other existing pain locations, self-reported comorbid conditions, and other relevant medical issues.

The follow-up questionnaires included the following clinical outcomes: SPADI, health-related quality of life (EQ-5D-5L), sleep quality (Sleep Quality Scale), pain intensity using Visual Analogue Scale (VAS), side effects, perceived recovery using the global perceived effect questionnaire (GPE), and adverse events.

#### Cost variables

The costs included in this study consisted of intervention costs, healthcare costs, informal care costs, absence of work costs, and unpaid productivity costs, measured using the the iMTA Medical Consumption Questionnaire (MCQ) [13] and the iMTA Productivity Cost Questionnaire (PCQ) [14]. The costs were measured at 6 weeks and 3, 6, 9, and 12 months. These MCQ and PCQ included the following cost outcomes:

- *Intervention costs*: this included the costs of the randomized interventions. In this case, the costs of the exercise therapy sessions and the corticosteroid injection(s).
- *Healthcare costs*: participants were asked to report the number of appointments with physiotherapists, general practitioners, social workers, occupational therapists, occupational physicians, medical specialists, and the medication used for their shoulder pain. It also included questions about whether someone had visited the emergency room, needed an ambulance, had to spend the night in the hospital, received healthcare at home, or was admitted to an institution, such as a rehabilitation center, due to their shoulder pain.
- *Informal care costs*: this included the care by family members, friends, and other volunteers and was valued at the recommended Dutch shadow price of 18.80 euro/h.
- *Absenteeism and presentism work costs*: Participants were asked to report the number of days one was absent from work because of their shoulder pain. Using the friction cost approach, with a friction period of 115 days, the total costs of absenteeism were valued. Participants were also asked to report whether they experienced productivity loss due to shoulder pain when they were working (presenteeism) on a scale of 0–10.
- *Unpaid productivity costs*: other activities and volunteer work participants were not able to do because of their shoulder pain. These hours were valued using the recommended Dutch shadow price of 18.80 euro/h.

The costs of these outcomes were based on the prices provided by the National Health Care Institute of the Netherlands (see Table 1) [20]. Medication costs were based on the prices provided by the Royal Dutch Society for Pharmacy [21].

**Table 1.** Healthcare costs based on the National Health Care Institute of the Netherlands [20].

	Costs (in euros)	Units
**Consultations**		
General practitioner	30.87	Per visit
Physiotherapist	38.89	Per visit
Occupational therapist	24.32	Per visit
Social worker	127.00	Per visit
Company doctor	120.00	Per visit
**Hospital costs**		
Outpatient clinic	120.00	Per visit
Day treatment	335.00	Per day
Emergency department	258.00	Per visit
Ambulance ride	528.00	Per ride
Admitted to the hospital	335.00	Per day
X-ray	82.17	Per test
MRI	271.00	Per test
Ultrasound	103.00	Per test
**Other**		
Staying elsewhere (e.g. residential care center)	849.00	Per day
Home care: Household activities	57.58	Per hour
Home care: Personal care	32.76	Per hour
Home care: Nursing	75.00	Per hour
Assistance from a friend or family member	18.80	Per hour
Incapacity to work	39.88	Per hour
Work productivity loss	18.80	Per hour

### Sample size

The sample size calculation for this trial was based on the clinical effectiveness outcome, the SPADI total score, since that was the primary outcome. Based on the most conservative estimate of variability in SPADI score (SD 20) [22], a sample size of 85 patients per group was estimated to be needed to achieve 90% power to detect a 10-point difference at a 5% two-tailed significance level. Prior to starting this trial, a loss to follow-up of 20% was considered, resulting in a required sample size of 213. However, during data collection, it became evident that a loss to follow-up of 15% was more accurate. Consequently, we recalculated the required sample size and adjusted the study protocol accordingly. This revised protocol was accepted in September 2024. Considering a 15% loss to follow-up, a total of at least 200 patients (100 per group) was needed.

### Missing data

Participants who only filled in one questionnaire were excluded from the analyses. Missing QALY data were imputed using multiple imputation by chained equations. The covariates used for multiple imputation included age, gender, baseline QALYs, and baseline SPADI scores based on clinical relevance and their correlation with outcomes. Predictive mean matching with 10 nearest neighbors was employed for imputation. Rubin's rules were applied to combine parameter estimates and standard errors across the 14 imputed datasets. A random seed, 12345, was used for reproducibility.

### Statistical methods

Baseline characteristics of the participants were summarized using descriptive statistics. The mean and standard deviation were reported for continuous variables, and the count and percentage were reported for the categorical variables. A difference of 10% or more between the injection group and the exercise therapy group would indicate a relevant difference.

The primary outcome for the cost-effectiveness analysis was the incremental cost-effectiveness ratio (ICER), defined as the difference in mean costs between the exercise therapy group and the injection group, divided by the difference in mean effectiveness:


$$ICER\;=\;\frac{\Delta \;Costs}{\Delta \;QALYs}$$

Costs were collected from a societal perspective and measured in euros, while effectiveness was measured in QALYs, derived from EQ-5D-5L scores obtained at baseline and follow-up.

To account for sampling uncertainty, non-parametric bootstrapping with 10 000 replications was performed. This involved taking the original data and creating 10 000 new datasets by repeatedly sampling from the original data with replacement, simulating different possible outcomes. For each of the 10 000 bootstrap samples, incremental costs, incremental QALYs, and ICERs were calculated.

The results were used to generate a cost-effectiveness plane and a cost-effectiveness acceptability curve (CEAC). The cost-effectiveness plane shows the relationship between incremental costs and QALYs for all 10 000 bootstrap samples. The CEAC shows the probability of cost-effectiveness at varying willingness-to-pay (WTP) thresholds. The WTP threshold per QALY in the Netherlands ranges from €20 000 to €80 000 and is based on disease severity. For this study, a WTP threshold of €50 000 was used, which is common for musculoskeletal complaints [23].

The CEAC was based on the proportion of bootstrap samples where the net monetary benefit (NMB) exceeded zero. The NMB calculates the value of the treatment, taking both its costs and health benefits into account and was calculated as: NMB=(WTP×ΔQALYs)−ΔCosts. If the NMB is positive, it indicates a cost-effective treatment as compared to the control.

## Results

The recruitment of participants was from February 2021 to August 2023, and data collection was completed in September 2024. A total of 200 participants were included in the SIX Shoulder Study, but 17 participants who only completed the baseline questionnaire were excluded from this analysis. Of the remaining 183 participants, 92 were randomized for the exercise therapy group and 91 for the corticosteroid injection group. Supplementary Appendix S1 shows a flow diagram of inclusion and follow-up.

### Descriptive statistics

Table 2 shows the baseline characteristics. The total group had a mean (± SD) age of 61 ± 12 years, with 53% being female. The mean baseline SPADI total score was 57 ± 20, and the mean duration of complaints was 34 ± 65 weeks, with 48% of the participants reporting pain for 3 months or less. There were no relevant differences (exceeding 10%) between the exercise therapy and the injection group at baseline.

**Table 2.** Descriptive statistics of participants of the SIX Shoulder Study.

*Characteristics*	Exercise therapy (*n* = 92)	Injection (*n* = 91)	Total (*n* = 183)
**Sex (%)**			
Female	49 (54)	48 (53)	97 (53)
Male	43 (46)	43 (47)	86 (47)
**Age ± SD**	61 ± 12	62 ± 12	61 ± 12
**BMI ± SD**	28 ± 5	27 ± 5	28 **±** 5
**Education, n (%)**			
Low	31 (34)	34 (37)	65 (36)
Middle	36 (39)	31 (34)	67 (37)
High	25 (27)	26 (29)	51 (28)
**Occupation, n (%)**			
Student	0 (0)	1 (1)	1 (1)
Paid laborer	48 (52)	42 (46)	100 (49)
Unemployed	12 (13)	17 (19)	29 (16)
**Retired**	32 (35)	31 (34)	63 (34)
Origin pain, n (%)			
Spontaneous	57 (62)	53 (58)	110 (60)
Sports	2 (2)	1 (1)	3 (2)
Work/hobby	18 (20)	19 (21)	37 (20)
Other	15 (16)	18 (20)	33 (18)
**Diagnosis, n (%)**			
Glenohumeral complaints	23 (25)	24 (26)	47 (26)
Subacromial pain syndrome	60 (65)	60 (66)	120 (66)
Missing	9 (10)	7 (8)	16 (9)
**Duration pain, mean weeks ± SD**	33 ± 65	34 ± 64	34 ± 65
**Use of painkillers, n (%)**	41 (45)	45 (50)	86 (47)
**Recurrent pain same shoulder, n (%)**	26 (28)	25 (27)	51 (28)
Received treatment^a^	19 (21)	23 (25)	42 (23)
Rest	1 (1)	1 (1)	2 (1)
Medication	2 (2)	3 (3)	5 (3)
Physiotherapy	13 (14)	11 (12)	24 (13)
Injection	11 (12)	12 (13)	23 (13)
Orthopedist	2 (2)	1 (1)	3 (2)
Rheumatologist	1 (1)	1 (1)	2 (1)
**Comorbidity, n (%)** ^a^			
Asthma/COPD	13 (14)	7 (8)	20 (15)
Myocardial infarction	4 (4)	2 (2)	6 (5)
Other cardiac disease	3 (3)	1 (1)	4 (3)
Stroke	6 (7)	2 (2)	8 (6)
Thyroid disease	4 (4)	0 (0)	4 (2)
Skin disease	2 (2)	8 (9)	10 (5)
Rheumatism	4 (4)	6 (7)	10 (5)
Other joint inflammation	13 (14)	12 (13)	25 (14)
Neck pain	36 (39)	30 (33)	66 (36)
Osteoarthritis	33 (36)	27 (30)	60 (33)
Psychological complaints	10 (11)	6 (7)	16 (9)
Consequence of accident	5 (5)	5 (5)	10 (5)
Median number of comorbidity	1	1	1
**FABQ**			
Pain	16 ± 5	15 ± 5	16 ± 5
Work	10 ± 12	9 ± 12	10 ± 12
HADS			
Fear	3 ± 4	3 ± 3	3 ± 3
Depression	3 ± 3	4 ± 3	4 ± 3
**Mean VAS ±SD**	75 ± 17	71 ± 18	73 ± 18
**Mean EQ-5D-5L ± SD**	0.7 ± 0.2	0.7 ± 0.2	0.7 ± 0.2
**Mean SPADI ±SD**	57 ± 20	57 ± 20	57 ± 20
Pain	63 ± 18	63 ± 18	63 ± 18
Function	51 ± 23	51 ± 24	51 ± 24

BMI, body mass index; COPD, chronic obstructive pulmonary disease; FABQ, Fear-Avoidance Beliefs Questionnaire; HADS, Hospital Anxiety and Depression Scale; SD, standard deviation; SPADI, Shoulder Pain and Disability Index; VAS, Visual Analogue Scale.

^a^Multiple responses per participant were permitted.

### Missing data

The dataset included 183 participants with complete cost data and 157 participants with complete EQ-5D-5L data at 12 months, leaving 26 participants (14.2%) with missing EQ-5D-5L values. After multiple imputation [24], the imputation diagnostics confirmed a monotone missing data pattern, allowing for efficient imputation without additional iterations. The final imputed dataset comprised 157 complete observations and 26 imputed observations in each of the 14 datasets.

### Costs

Table 3 shows the total costs and mean costs per patient per treatment group. The mean number of exercise therapy sessions was 10.9 in the exercise therapy group and 3.9 in the injection group. The mean number of injections was 0.37 in the exercise therapy group and 1.3 in the injection group. The total healthcare costs were €113 093.11 for the exercise therapy group and €139 545.64 for the injection group, with a significant portion of these costs attributed to assistance from friends or family. The productivity costs were, respectively, €350 041.45 and €174 201.92 for the exercise therapy group and the injection group.

**Table 3.** Overview of healthcare costs and productivity costs per treatment group.

	Exercise therapy (*n* = 92)			Injection group (*n* = 91)		
	Mean number of appointments ± SD	Cost per patient (Euro's)	Total costs (Euro's)	Mean number of appointments ± SD	Cost per patient (Euro's)	Total costs (Euro's)
**Healthcare use**						
Exercise therapy	10.9 ± 7.13	425.25	39 123.34	3.98 ± 7.16	153.02	14 078.18
Injection	0.37 ± 0.93	2.22	204	1.3 ± 1.03	7.76	714
General practitioner	1.76 ± 2.94	54.36	5000.94	2.55 ± 3.03	77.51	7130.97
Social worker	0.14 ± 1.06	17.95	1651	0.22 ± 1.40	27.61	2540
Occupational therapist	0.18 ± 1.28	4.49	413.44	0.01 ± 0.10	0.26	24.32
Company doctor	0.30 ± 0.97	36.52	3360	0.07 ± 0.44	7.83	720
Hospital costs		55.73	5127		77.97	7173.51
Home care		91.19	8389.56		98.74	9084
Medication		11.23	1032.79		12.99	1195.98
Help from friends or family		530.34	48 791.04		1053.10	96 884.86
Total healthcare costs			113 093.11			139 545.64
**Productivity costs**						
Absenteeism		2765.81	254 454.9		723.76	66 585.93
Presenteeism		725.31	66 728.55		597.77	54 994.79
Unpaid productivity loss		313.67	28 858		571.97	52 621.20
Total productivity costs			350 041.45			174 201.92

SD, standard deviation.

### Cost-effectiveness

**Figure 1.**
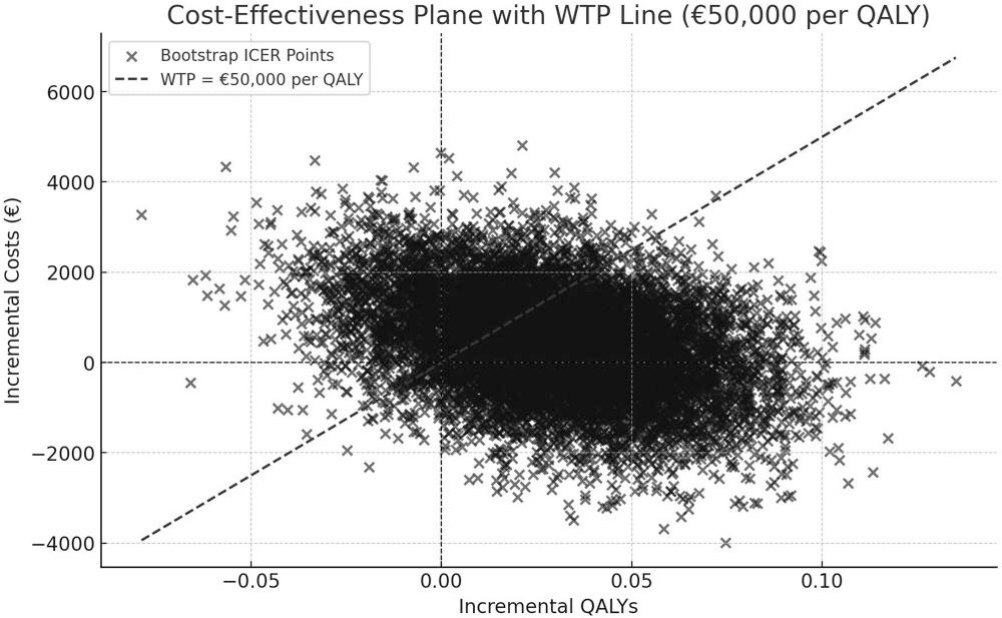
Bootstrap scatterplot with 10 000 replications representing the cost-effectiveness at a WTP-threshold of €50 000 per QALY of patients with shoulder pain in primary care (2021–2024). QALY, quality-adjusted life years; WTP, willingness-to-pay threshold.

The mean total cost for the exercise therapy and the injection group was €3446 and €3018, respectively. The mean QALYs for the exercise therapy and the injection group were 0.8095 and 0.7799, respectively. The incremental costs for the exercise therapy group compared to the injection group over 12 months were €428 (95% CI: −€1825 to €2682). The incremental QALY was 0.0296 (95% CI: −0.0299 to 0.0891). The ICER was calculated as €14 489 (95% CI: −1 698 053 to 1 727 032) per QALY gained (Table 4), indicating that for each QALY gained from the exercise therapy group compared to the injection group, the costs were €14 489. Bootstrapping produced a 95% confidence interval of −€348 881 to €314 053, highlighting substantial uncertainty. The cost-effectiveness plane (Fig. 1) showed most bootstrap samples in the northeast quadrant, indicating that exercise therapy provided additional health benefits, but at a higher cost. A significant proportion of points fall below the WTP line, suggesting that exercise therapy has a high probability of being cost-effective at €50 000 per QALY.

**Table 4.** Cost-effectiveness outcomes.

Outcome	Mean	Standard error	95% CI
**Incremental costs**	€428	1142	−1825 to 2682
**Incremental QALYs**	0.02957	0.03011	−0.0299 to 0.0891
**ICER**	€14 489	873 762	−1 698 053 to 1 727 032

CI, confidence interval; ICER, incremental cost-effectiveness ratio; QALY, quality-adjusted life years.

**Figure 2.**
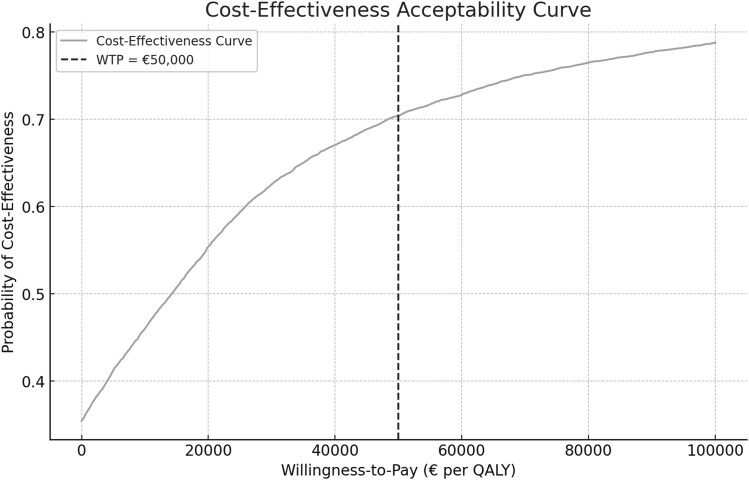
Cost-effectiveness acceptability curve of patients with shoulder pain in primary care (2021–4).

The CEAC (Fig. 2) showed that the probability of cost-effectiveness was ∼70% at a WTP threshold of €50 000 per QALY. This indicates that there is a 70% chance that exercise therapy is cost-effective when society is willing to pay €50 000 for an additional QALY.

At a WTP threshold of €50 000 per QALY, the mean NMB for exercise therapy was €1077 (95% CI: €1037–1117). One sample *t*-test confirmed the NMB was significantly greater than zero (*P* < 0.001), supporting the cost-effectiveness of exercise therapy at this threshold (Supplementary Appendix S3, Table 1). At a WTP of €50 000 per QALY, the NMB histogram showed that most values of the bootstrap samples are between €500 and €2000, indicating a high probability of positive net benefit (Supplementary Appendix S4, Fig. 1).

Supplementary Appendices S5 and S6 show the histograms of incremental costs and QALYs, where both distributions were approximately normal. Incremental costs were centered around €0, indicating that the cost difference between exercise therapy and the injection group was on average close to zero. Additionally, incremental QALYs were slightly positive, indicating that on average the exercise therapy group provided a small health benefit compared to the injection group.

## Discussion

This study examined the cost-effectiveness of exercise therapy compared to a corticosteroid injection in patients with shoulder pain over a 12-month follow-up. The results indicated that on average the exercise therapy group provided a small health benefit compared to the injection group with a WTP of 50 000 euros (ICER = €14 489, 95% CI: −1 698 053 to 1 727 032). There is a 70% chance that the exercise therapy group is cost-effective compared to the injection group, when society is willing to pay 50 000 euros for an additional QALY. A large proportion of bootstrap samples fall below the €50 000 per QALY WTP line, indicating that the intervention is cost-effective under this threshold in most cases. However, the presence of points in the northwest quadrant (positive costs, negative QALYs) suggests that there is considerable uncertainty in the cost-effectiveness estimates.

This study is the first to compare the cost-effectiveness of two commonly used treatments for general shoulder pain in primary care, corticosteroid injections and exercise therapy, over a 12-month follow-up. Several studies investigated general costs of shoulder pain in primary healthcare [8, 25], but none compared these treatments for shoulder pain. Nevertheless, the same trend of results is seen in other musculoskeletal complaints comparing exercise therapy with a corticosteroid injection [26–28]. For example, the study of Rhon *et al* [26]. investigated these treatment options for knee osteoarthritis. They also suggested that although exercise therapy is more expensive, the greater improvement of quality-adjusted life-years is worth the additional cost over a 12-month follow-up.

The results of this study showed uncertainty, potentially attributable to significant variability in the total costs per individual. Notably, 16.4% of participants accounted for 74% of the costs. These costs were mainly due to sick leave or productivity losses. However, similar patterns have been observed in other cost-effectiveness trials and are not unusual in the context of musculoskeletal disorders [8, 25].

One limiting factor is the number of missing data, with 14% missing EQ-5D-5L data at 12 months. However, multiple imputation is an appropriate and widely used method of imputing the missing data, to prevent excluding too many participants due to missing data of the outcome [24].

Another limitation was the use of self-reported questionnaires. While this is very valuable for collecting data from participants, they come with several drawbacks [29]. First of all, participants may provide socially desirable responses regarding their healthcare use, which can lead to overreporting of positive behavior and underreporting of the negative ones. Second, self-reported questionnaires often depend on the memory of the participants, which can be unreliable, resulting in recall-bias. Also, the self-reported questionnaires resulted in missing data, since some questions were either answered incorrectly or left blank. Lastly, despite mentioning that participants should only report their healthcare usage and absenteeism regarding their shoulder pain, there is a realistic possibility that participants reported all healthcare use and absenteeism, and not only for their shoulder pain.

One strength of this study was the fact that the sample size of 200 participants was met. The baseline table also shows that the randomization was successful, since there were no relevant differences between the two groups.

Another strength is that we used both the societal and medical perspectives, making sure it was possible to draw a conclusion based on both aspects. Also, the cooperation of many GP practices and the pragmatic design adds to the generalizability of the results in the Dutch population.

Although the incremental costs for the exercise therapy group were higher with 428 euros compared to the injection group, this study showed that exercise therapy is still likely to be more cost-effective compared to an injection over a 12-month follow-up at a WTP threshold of €50 000 per QALY. We can conclude that for patients with shoulder pain, exercise therapy is recommended as the preferred course of treatment.

Future research with the data from this trial will focus on the effectiveness of the two treatments. Other future research could focus on patient preferences for the treatment options.

## Conclusion

This RCT investigated the cost-effectiveness of a corticosteroid injection versus exercise therapy in patients with shoulder pain over 12 months. Although the data showed uncertainty, the study's main result showed that for patients with shoulder pain in primary care, the exercise therapy group is likely to be more cost-effective compared to the injection group over a 12-month follow-up with an ICER of €14 489 (95% CI: −1 698 053 to 1 727 032) at a WTP threshold of €50 000 per QALY.

## Supplementary Material

cmaf081_Supplementary_Data

## Data Availability

The data underlying this article will be shared on reasonable request to the corresponding author.
