# Supplementary material for: Cost-effectiveness of a corticosteroid injection versus exercise therapy for shoulder pain in general practice (SIX-Shoulder Study): a randomized controlled trial
Source: Fam Pract. 2025 Nov 17;42(6):cmaf081. doi: 10.1093/fampra/cmaf081 (PMC12620349; doi:10.1093/fampra/cmaf081)
Supplement: cmaf081_Supplementary_Data [file cmaf081_supplementary_data.pdf]

APPENDIX 1: FLOWDIAGRAM

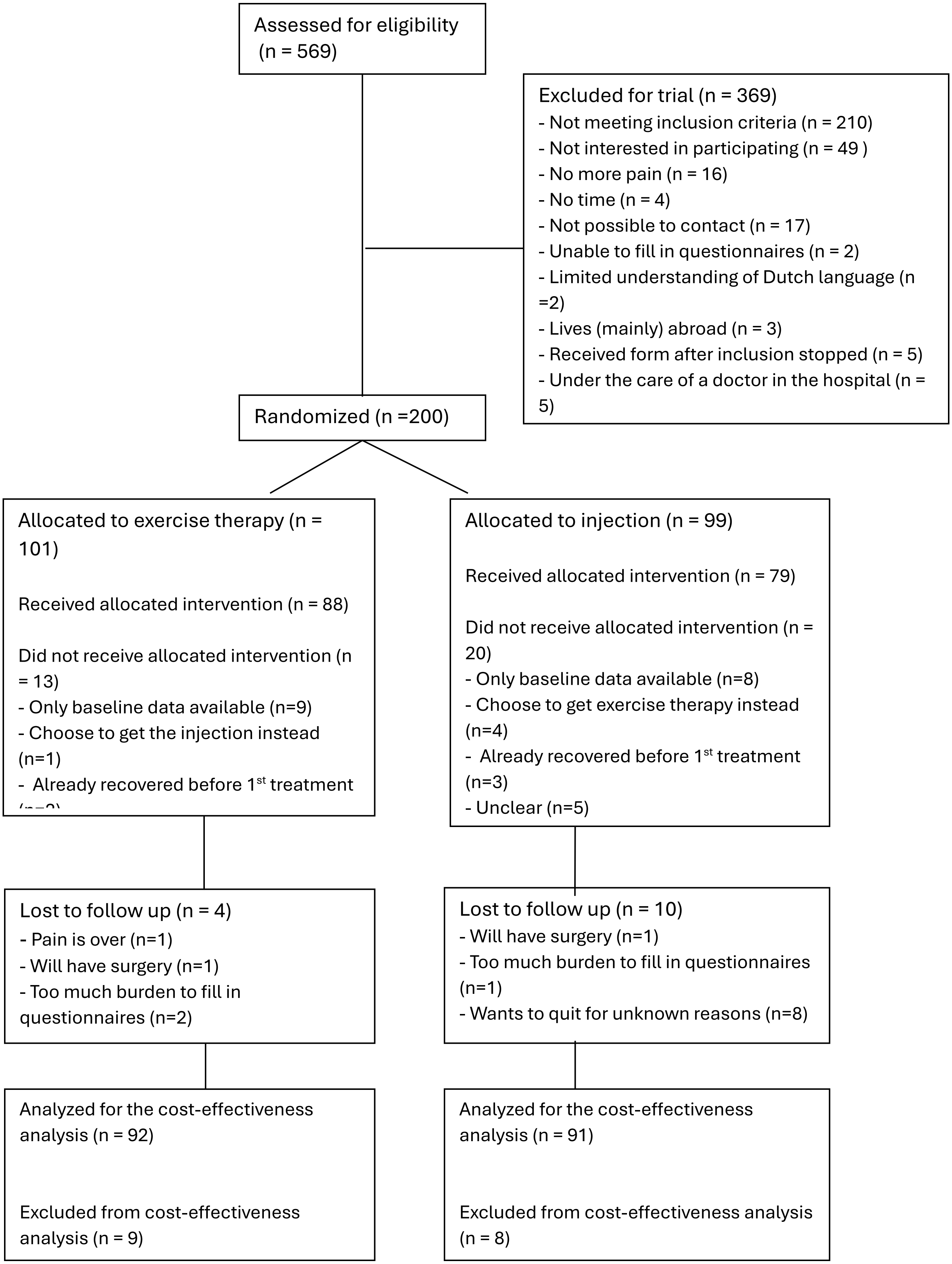

APPENDIX 2: Short guidelines for participating physiotherapists

INTRODUCTION

You are receiving this guideline as a result of your patient's participation in the SIX Shoulder Study, a randomized trial investigating the treatment of patients with shoulder complaints. This study is a pragmatic effectiveness trial initiated by Erasmus MC and compares the (cost-)effectiveness of a local corticosteroid injection versus exercise therapy in patients experiencing a new episode of shoulder complaints. A total of 213 patients will be randomized to receive one of the two treatments.

The NHG guideline 'Shoulder Complaints' advises a stepwise treatment approach for patients with glenohumeral joint complaints or subacromial pain syndrome (SAPS). If symptoms do not sufficiently improve after education, advice, and pain relief, the general practitioner may consider the option of administering a local corticosteroid injection or referring the patient for exercise therapy.

It is currently unknown which treatment is superior in the long term. This study aims to provide a scientifically substantiated choice for the general practitioner between the two treatments.

This guideline is intended to outline the framework for the exercise therapy program, so that a definitive conclusion can be drawn about the effectiveness of exercise therapy in patients with shoulder complaints. You are expected to adhere to the guidelines described herein**. If you wish to deviate from this guideline for your patient, please contact the research team (see contact details).** This guideline was developed in collaboration with the Shoulder Network Netherlands (SNN) and is based on recommendations from scientific research.

CRITERIA OF THE EXERCISE PROGRAM

Duration and frequency

The aim is for participating patients to receive a maximum of 12 treatments over a period of 12-14 weeks. Each treatment session lasts an average of 30 minutes per visit. In addition to the regular treatment, it is intended that the patient engages in home exercises at least 3 times per week.

Intensity

For the intensity of the exercises to be performed, it is recommended to use the Rating of Perceived Exertion (RPE) scale from 0 to 10 points, as scored by the patient.

Rating of perceived exertion (RPE 0 – 10)										
0	1	2	3	4	5	6	7	8	9	10
Rest	Really easy	Easy	Moderate	Challenging	Hard		Really hard		Really, really, hard	Maximal

The intensity should be adjusted to the tissue's load-bearing capacity and the patient's condition. A (post-) reaction of pain from the exercises is permissible, as long as there is no nocturnal pain and the pain subsides to the pre-training level within 24 hours.

Additional Interventions

Mobilization and/or manipulation techniques can be used alongside the exercise program to achieve the desired treatment goals. Massage, laser therapy, ultrasound, TENS, acupuncture, ESWT, and dry needling are discouraged.

Extension of the Exercise Therapy Program

If you need to extend the exercise therapy program for your patient for any reason, please inform the SIX research team.

Termination of the Exercise Therapy Program

The treatment can be discontinued if, according to the physiotherapist and the patient, the treatment objectives have been met. If the patient needs to stop the exercise therapy for other reasons, it is recommended that the patient personally contacts the SIX research team (see contact details) to discuss the situation.

GOAL OF THE EXERCISE THERAPY PROGRAM

The goal of physiotherapeutic guidance is aimed at reducing the health problem, considering biological, psychological, and social factors that may play a role and lead to the establishment of the following treatment goals:

- Improving impairments in functions, such as pain, movement limitation, or strength
- Building up activities and participation
- Optimizing movement patterns
- Increasing the patient's knowledge and understanding
- Promoting an adequate way of managing the complaints

In addition to providing exercise therapy, the physiotherapist has an important role in increasing the patient's knowledge and understanding, thereby promoting an adequate way of managing the complaints. Alleviating and preventing anxiety and uncertainty about the condition and paying attention to contextual factors are also important for preventing negative health beliefs and thereby reducing unnecessary and undesirable negative stress factors. Exercise therapy and hands-on techniques can be used by the physiotherapist to improve the patient's confidence in their shoulder and thereby create a more positive expectation. The physiotherapist guides the patient and encourages them to maintain and expand their progress in movement behavior to full activities and participation.

STRUCTURE OF THE EXERCISE THERAPY PROGRAM

For the structure of the exercise therapy program, a phasing is recommended based on the reactivity of the tissue to be loaded and the load-bearing capacity of the patient. The table below can be used as a guideline for building up the exercise therapy program.

Table 1: Structure of the exercise therapy program with 3 phases

	**Phase 1 – High reactivity**	**Phase 2 – Moderate reactivity**	**Fase 3 – Low reactivity**
**Characteristics**<br><br>** If applicable*	High pain level (NPRS >=7)<br>Frequent night pain and/or rest pain<br>Significant functional limitations<br>Resistance testing +++, very painful, can generate little strength*<br>Pain before the end of the Range of Motion (ROM)*<br>*AROM < PROM**	Moderate pain level (NPRS 4-6)<br>Occasional night pain and/or rest pain<br>Moderate functional limitations<br>Resistance testing ++, provokes pain and decreased strength*<br>Pain at the end of the Range of Motion (ROM)*<br>*AROM ~ PROM**	Low pain level (NPRS <=3) No night pain and/or rest pain<br>Mild functional limitations<br>Resistance testing +, mildly painful, less strength than healthy side*<br>Minimal pain at end of ROM*<br>*AROM = PROM**
**Focus of the exercise therapy program**	Isometric exercises<br>Neuromuscular coordination<br>Improve movement pattern<br>Improve posture	Concentric exercises<br>Eccentric exercises<br>Start functional exercises<br>Start return to work	Functional exercises<br>Plyometric exercises<br>Return to work<br>Return to sport
**Intensity**	Low intensity (RPE 2-4)	Moderate intensity (RPE 4-6)	Moderate to high intensity (RPE 4-8)

APPENDIX 3: T-test NMB

Table S1. T-test Net Monetary Benefit considering the WTP threshold of €50,000 per QALY

Variable	Observations	Mean	Standard Error	Standard Deviation	95% CI
NMB 50,000	10,000	1077.25	20.53	2,052.66	1,037.01 to 1,117.48

APPENDIX 4: Histogram for Net Monetary Benefit Distribution

Figure 1: histogram of the Net Monetary Benefit distribution

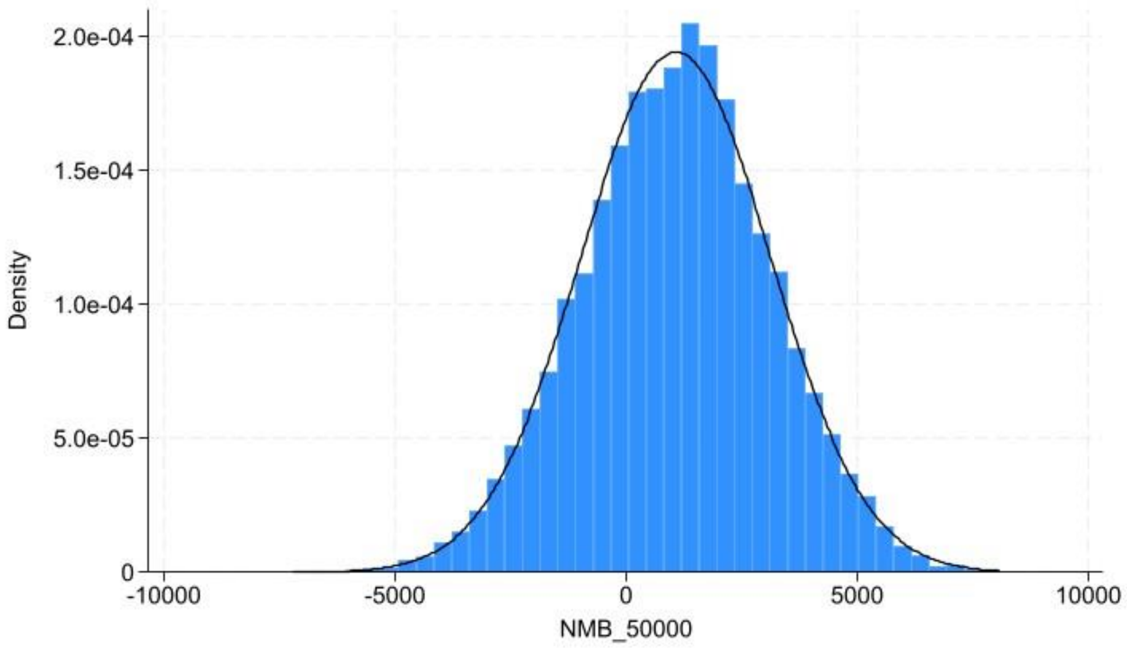

APPENDIX 5: Histogram for Incremental Costs Distribution

Figure 1: histogram of the Incremental Costs distribution

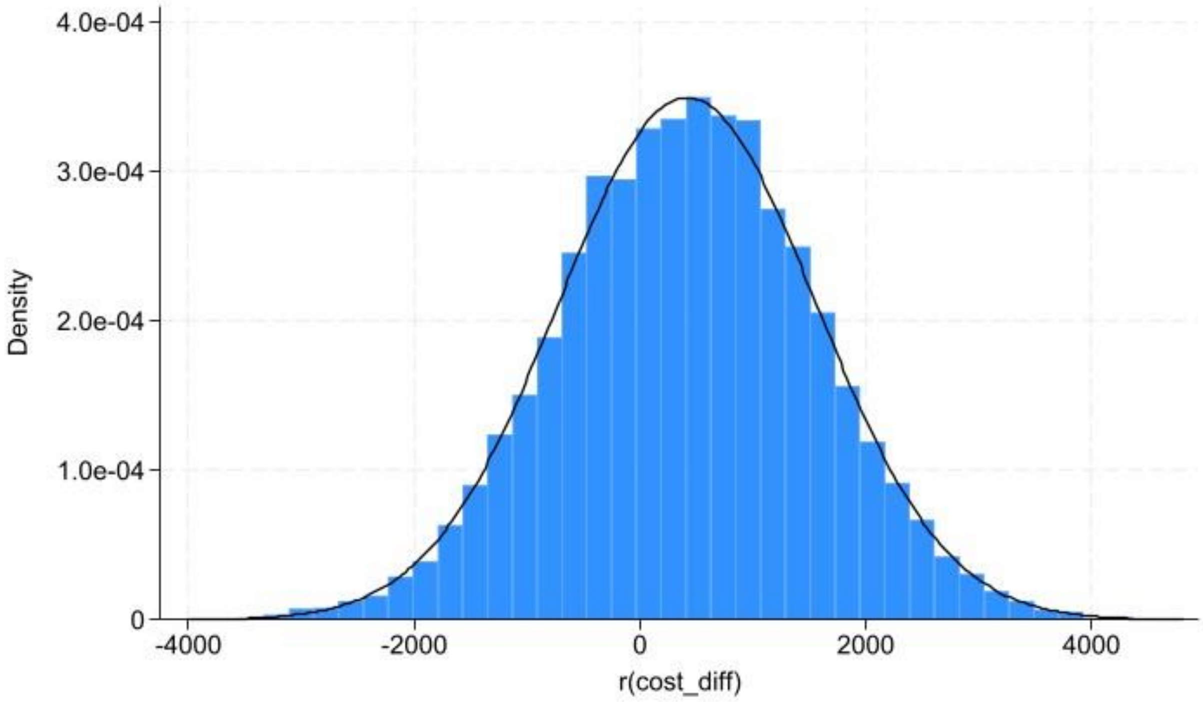

APPENDIX 6: Histogram for Incremental QALYs Distribution

Figure 1: histogram of the Incremental QALYs distribution

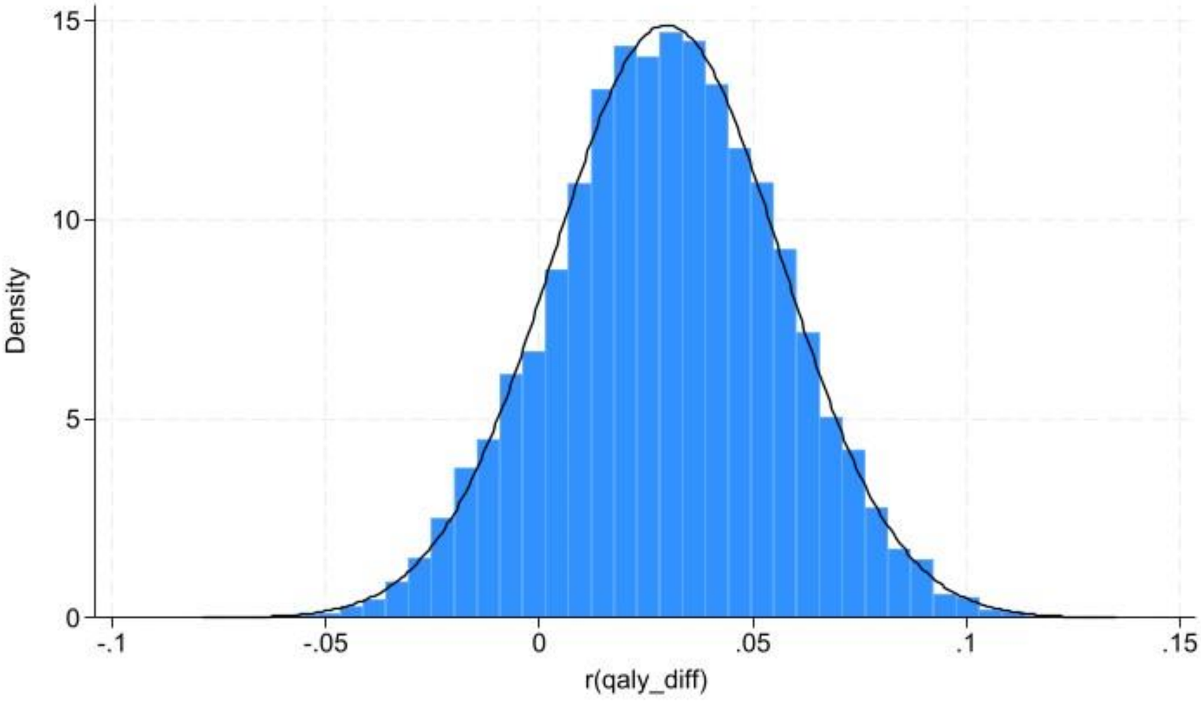

CONSORT 2010 checklist of information to include when reporting a randomised trial*

Section/Topic	Item No	Checklist item	Reported on page No
**Title and abstract**			
	1a	Identification as a randomised trial in the title	1
	1b	Structured summary of trial design, methods, results, and conclusions (for specific guidance see CONSORT for abstracts)	2
**Introduction**			
Background and objectives	2a	Scientific background and explanation of rationale	4
	2b	Specific objectives or hypotheses	4
**Methods**			
Trial design	3a	Description of trial design (such as parallel, factorial) including allocation ratio	5
	3b	Important changes to methods after trial commencement (such as eligibility criteria), with reasons	9
Participants	4a	Eligibility criteria for participants	5
	4b	Settings and locations where the data were collected	5
Interventions	5	The interventions for each group with sufficient details to allow replication, including how and when they were actually administered	6
Outcomes	6a	Completely defined pre-specified primary and secondary outcome measures, including how and when they were assessed	6-8
	6b	Any changes to trial outcomes after the trial commenced, with reasons	-
Sample size	7a	How sample size was determined	9
	7b	When applicable, explanation of any interim analyses and stopping guidelines	-
Randomisation:			
Sequence generation	8a	Method used to generate the random allocation sequence	5
	8b	Type of randomisation; details of any restriction (such as blocking and block size)	5
Allocation concealment mechanism	9	Mechanism used to implement the random allocation sequence (such as sequentially numbered containers), describing any steps taken to conceal the sequence until interventions were assigned	5
Implementation	10	Who generated the random allocation sequence, who enrolled participants, and who assigned participants to interventions	5
Blinding	11a	If done, who was blinded after assignment to interventions (for example, participants, care providers, those	5

		assessing outcomes) and how	
	11b	If relevant, description of the similarity of interventions	-
Statistical methods	12a	Statistical methods used to compare groups for primary and secondary outcomes	9-10
	12b	Methods for additional analyses, such as subgroup analyses and adjusted analyses	9-10
**Results**			
Participant flow (a diagram is strongly recommended)	13a	For each group, the numbers of participants who were randomly assigned, received intended treatment, and were analysed for the primary outcome	10-11, 21
	13b	For each group, losses and exclusions after randomisation, together with reasons	21
Recruitment	14a	Dates defining the periods of recruitment and follow-up	10
	14b	Why the trial ended or was stopped	-
Baseline data	15	A table showing baseline demographic and clinical characteristics for each group	11
Numbers analysed	16	For each group, number of participants (denominator) included in each analysis and whether the analysis was by original assigned groups	12
Outcomes and estimation	17a	For each primary and secondary outcome, results for each group, and the estimated effect size and its precision (such as 95% confidence interval)	14
	17b	For binary outcomes, presentation of both absolute and relative effect sizes is recommended	-
Ancillary analyses	18	Results of any other analyses performed, including subgroup analyses and adjusted analyses, distinguishing pre-specified from exploratory	14-15
Harms	19	All important harms or unintended effects in each group (for specific guidance see CONSORT for harms)	-
**Discussion**			
Limitations	20	Trial limitations, addressing sources of potential bias, imprecision, and, if relevant, multiplicity of analyses	16-17
Generalisability	21	Generalisability (external validity, applicability) of the trial findings	17
Interpretation	22	Interpretation consistent with results, balancing benefits and harms, and considering other relevant evidence	16
**Other information**			
Registration	23	Registration number and name of trial registry	2
Protocol	24	Where the full trial protocol can be accessed, if available	5
Funding	25	Sources of funding and other support (such as supply of drugs), role of funders	18

Citation: Schulz KF, Altman DG, Moher D, for the CONSORT Group. CONSORT 2010 Statement: updated guidelines for reporting parallel group randomised trials. BMC Medicine. 2010;8:18. © 2010 Schulz et al. This is an Open Access article distributed under the terms of the Creative Commons Attribution License (http://creativecommons.org/licenses/by/2.0), which permits unrestricted use, distribution, and reproduction in any medium, provided the original work is properly cited.

*We strongly recommend reading this statement in conjunction with the CONSORT 2010 Explanation and Elaboration for important clarifications on all the items. If relevant, we also recommend reading CONSORT extensions for cluster randomised trials, non-inferiority and equivalence trials, non-pharmacological treatments, herbal interventions, and pragmatic trials. Additional extensions are forthcoming: for those and for up-to-date references relevant to this checklist, see www.consort-statement.org.
